# Cigarette Smoke Induces C/EBP-β-Mediated Activation of miR-31 in Normal Human Respiratory Epithelia and Lung Cancer Cells

**DOI:** 10.1371/journal.pone.0013764

**Published:** 2010-10-29

**Authors:** Sichuan Xi, Maocheng Yang, Yongguang Tao, Hong Xu, Jigui Shan, Suzanne Inchauste, Mary Zhang, Leandro Mercedes, Julie A. Hong, Mahadev Rao, David S. Schrump

**Affiliations:** 1 Thoracic Oncology Section, Surgery Branch, Center for Cancer Research, National Cancer Institute, Bethesda, Maryland, United States of America; 2 Laboratory of Cancer Prevention, National Cancer Institute, Frederick, Maryland, United States of America; 3 Advanced Biomedical Computing Center, SAIC-Frederick, National Cancer Institute, Frederick, Maryland, United States of America; Helmholtz Zentrum München/Ludwig-Maximilians-University Munich, Germany

## Abstract

**Background:**

Limited information is available regarding mechanisms by which miRNAs contribute to pulmonary carcinogenesis. The present study was undertaken to examine expression and function of miRNAs induced by cigarette smoke condensate (CSC) in normal human respiratory epithelia and lung cancer cells.

**Methodology:**

Micro-array and quantitative RT-PCR (qRT-PCR) techniques were used to assess miRNA and host gene expression in cultured cells, and surgical specimens. Software-guided analysis, RNA cross-link immunoprecipitation (CLIP), 3′ UTR luciferase reporter assays, qRT-PCR, focused super-arrays and western blot techniques were used to identify and confirm targets of miR-31. Chromatin immunoprecipitation (ChIP) techniques were used to evaluate histone marks and transcription factors within the LOC554202 promoter. Cell count and xenograft experiments were used to assess effects of miR-31 on proliferation and tumorigenicity of lung cancer cells.

**Results:**

CSC significantly increased miR-31 expression and activated LOC554202 in normal respiratory epithelia and lung cancer cells; miR-31 and LOC554202 expression persisted following discontinuation of CSC exposure. miR-31 and LOC554202 expression levels were significantly elevated in lung cancer specimens relative to adjacent normal lung tissues. CLIP and reporter assays demonstrated direct interaction of miR-31 with Dickkopf-1 (Dkk-1) and DACT-3. Over-expression of miR-31 markedly diminished Dkk-1 and DACT3 expression levels in normal respiratory epithelia and lung cancer cells. Knock-down of miR-31 increased Dkk-1 and DACT3 levels, and abrogated CSC-mediated decreases in Dkk-1 and DACT-3 expression. Furthermore, over-expression of miR-31 diminished SFRP1, SFRP4, and WIF-1, and increased Wnt-5a expression. CSC increased H3K4Me3, H3K9/14Ac and C/EBP-β levels within the LOC554202 promoter. Knock-down of C/EBP-β abrogated CSC-mediated activation of LOC554202. Over-expression of miR-31 significantly enhanced proliferation and tumorigenicity of lung cancer cells; knock-down of miR-31 inhibited growth of these cells.

**Conclusions:**

Cigarette smoke induces expression of miR-31 targeting several antagonists of cancer stem cell signaling in normal respiratory epithelia and lung cancer cells. miR-31 functions as an oncomir during human pulmonary carcinogenesis.

## Introduction

Genetic as well as epigenetic dysregulation of gene expression during malignant transformation is attributable in part to aberrant expression of micro-RNAs (miRNAs) [1], [2]. These small (∼21-mer) non-coding RNA molecules regulate gene expression by binding to 3′ untranslated regions (3′ UTR) of target mRNAs, triggering transcript degradation or translational repression depending on the extent of complementarity between the seed sequence of the miRNA and the mRNA motif [3]. Approximately 30% of all mRNAs are potential miRNA targets. To date, more than 800 miRNAs have been identified in humans, each of which targets multiple functionally-related genes, thus mediating complex regulatory networks [3], [4].

A variety of miRNAs have been implicated in the pathogenesis of human lung cancers, the vast majority of which are directly attributable to cigarette smoking [5], [6]. Some of these miRNAs function as oncogenes (oncomirs), whereas others act as tumor suppressors. For example, miR-21, which is activated in part via aberrant EGFR signaling represses expression of PTEN and PDCD4, facilitating proliferation, invasion, and resistance to apoptosis of lung cancer cells [7]–[11]. Activation of miR-93, miR-98 and miR-197 inhibits expression of FUS1, enhancing cell cycle progression and chemo-resistance in lung cancer cells [12], [13]. Repression of miR-15A and miR-16, which target cyclins D1, D2 and E, abrogates Rb-mediated cell cycle regulation in lung cancer cells [14]. Furthermore, down-regulation of let-7 family members targeting numerous mRNAs encoding cell cycle regulatory proteins such as Ras, AURKA, AURKB, and E2F5 enhances proliferation and tumorigenicity of lung cancer cells [7], [15], [16]. Interestingly, several miRNA alterations frequently observed in lung cancers, such as down-regulation of let-7 and over-expression of miR-17-92, are detected in cancer stem cells [17], [18] as well as pseudoglandular lung parenchyma [19], suggesting embryonic reprogramming of miRNA expression during pulmonary carcinogenesis.

Despite recent studies demonstrating miRNA expression profiles correlating with tumor histology, as well as smoking status and prognosis of patients with primary lung cancers, [6], [20]–[22], limited information is available regarding miRNA alterations that directly contribute to initiation and early progression of these malignancies. In the present study, we utilized an in-vitro model system to examine miRNA alterations mediated by cigarette smoke condensate (CSC) in normal human respiratory epithelia, and lung cancer cells derived from smokers as well as nonsmokers. Herein, we report that CSC induces expression of miR-31 targeting several Wnt signaling antagonists including Dickkhopf-1 (Dkk-1) and DACT3 in normal human respiratory epithelia as well as lung cancer cells. These observations provide a direct mechanistic link between cigarette smoke and activation of an oncomir suppressing antagonists of stem cell signaling during tobacco-induced pulmonary carcinogenesis.

## Materials and Methods

### Cell lines and treatment conditions

All lung cancer lines were obtained from American Type Culture Collection (ATCC; Manassas, VA), and maintained in RPMI medium supplemented with 10% FCS, 10 mM of glutamic acid, and 1% penicillin/streptomycin. Primary normal human small airway epithelial cells (SAEC) and normal human bronchial epithelial cells (NHBE) were obtained from Lonza, Inc (Frederick, MD), and cultured per vendor instructions. Immortalized human bronchial epithelial cells (HBEC) were generously provided by John D. Minna (U-T Southwestern, Dallas, TX), and cultured in complete Keratinocyte-SFM media (Invitrogen, Carlsbad, CA) supplemented with 5 µg/L epidermal growth factor (EGF) and 50 mg/L bovine pituitary extract (BPE). Cigarette smoke condensates (CSC) derived from Kentucky Reference 3R4F research blend cigarettes (University of Kentucky) were prepared as described [23], and resuspended at a concentration of 1 mg tar/mL in RPMI, which was defined as 10% CSC [24]. For smoke exposure experiments, cells were cultured in 10-cm dishes in appropriate normal media (NM) with or without CSC (1%). Medium was changed daily with the addition of fresh CSC. Cells were subcultured as necessary, and harvested at various time-points for analysis.

### Human tissues

Primary lung cancer specimens and adjacent histologically normal lung parenchyma were harvested intra-operatively from patients undergoing potentially curative resections on NIH internal review board–approved protocols, requiring written informed consent. All tissues were immediately snap-frozen with a portion of harvested tissue sent for immediate histologic confirmation by an independent, anatomic pathologist in a blinded manner. Tissue specimens were bar-coded and stored in the Thoracic Oncology Laboratory, Surgery Branch, NCI. For microRNA isolation and expression analysis, the small RNA fraction was isolated with the RT2 qPCR-Grade miRNA Isolation kit (Qiagen; Valencia, CA), and miRNA expression was quantified with the qRT-PCR miRNA Detection Kit (ABI; Carlsbad, CA).

### miRNA overexpression and inhibition

Precursor and inhibitor miRNAs pMiR-H31PA-1, pCDH-CMV-MCS-EF1-copGFP negative control, MZIP31-PA-1, pGreenPuro Scramble Hairpin negative control were purchased from SBI (System Biosciences, Mountain View, CA). miRNA precursors or inhibitors (both at 50 nM) were transfected into cells using Lipofectamine 2000 (Invitrogen, Carlsbad, CA).

### Reporter vectors and DNA constructs

The 3′ UTRs of Dkk1 (850 bp) and DACT3(2461 bp) were PCR amplified from human SAEC cDNA, and inserted downstream of CMV-driven firefly luciferase cassette in the pMIR-REPORT vector (Ambion, Austin, TX) between *Hind III* and *Spe I* sites. Mutant reporter plasmids (3′ UTRs of Dkk1 and DACT3) were created by using QuikChange Site-Directed Mutagenesis Kit (Stratagene, Wilmington, DE). All insert fragments were confirmed by direct sequencing.

### Luciferase miRNA target reporter assays

For miRNA target validation, approximately 2×10^4^ SAEC cells per well in 24-well plates were transiently transfected with 25 to 50 ng of each firefly luciferase reporter construct (Promega, Madison, WI), 150 to 175 ng pcDNA3 empty vector, 200-ng pRTK-Luc (Promega) as internal control, and 30 pmol of pre-miR-31 (SBI, Mountain View, CA). Renilla luciferase vector was used to normalize transfection efficiency. Approximately 24 hours after transfection, firefly and Renilla luciferase activities were assayed. Normalized relative light units represent firefly luciferase activity/Renilla luciferase activity.

### Proliferation assays

Cells were plated at a concentration of 5×10^4^ cells per well in 24-well plates and cultured in NM with or without CSC plus plasmid transfection. Triplicate wells were harvested and counted by trypan blue exclusion techniques.

### Quantitative RT-PCR

For mRNA, total RNA was prepared using TRIzol reagent (Invitrogen) and genomic DNA was eliminated with TURBO DNA-free Kit (Ambion). One µg of total RNA was reverse transcribed using iScript reverse transcriptase (Bio-Rad). Omission of reverse transcriptase served as a negative control. cDNA was amplified using Platinum PCR SuperMix (Invitrogen). PCR was performed as follows: 5 min at 94°C, 35 cycles of 60 s at 94°C, 60 s at 57–60°C, and 60 s at 72°C, followed 5 min at 72°C. Real-time quantitative RT-PCR analysis was done as described [25] using Dkk-1, DACT3, SFRP1, SFRP4, WIF-1, and β-actin primers from Applied Biosystems or Integrated DNA Technologies (Coralville, IA). For microRNA isolation and expression analysis, expression of miRNA isolated with the RT2 qPCR-Grade miRNA Isolation kit (Qiagen) was quantified with the qRT-PCR miRNA Detection Kit (ABI).

### Chromatin immunoprecipitation (ChIP)

Cells were crosslinked with 1% formaldehyde, lysed and sonicated on ice to generate DNA fragments with an average length of 200–800 bp [26]. After pre-clearing, 1% of each sample was saved as input fraction. Immunoprecipitation was performed using antibodies specifically recognizing H3K4me3, H3K9Ac, H3K14Ac (Abcam), RNA polymerase II (Upstate) or IgG control. DNA was eluted and purified from complexes, followed by PCR amplification of the LOC554202 promoter using primers and conditions as described [27].

### siRNA and shRNA knockdown

Calu-6 and H841 cells were transiently transfected with siRNAs targeting C/EBP-α and C/EPB-β, or sham siRNA sequences (Dharmacon, Lafayette, CO) using Lipofectamine 2000 (Invitrogen). Target gene knockdown was confirmed by RT-PCR and western blot techniques.

### Western blot analysis

Protein extracts were generated as previously described [26]. Samples were separated on 4–12% Tris-glycine SDS-polyacrylamide gel electrophoresis gels and blotted onto Immobilon P membrane (Millipore, Billerica, MA); proteins were detected using enhanced chemiluminescence detection reagents (Amersham Biosciences, Piscataway, NJ). Antibodies used for western analysis were goat anti-Dkk-1, mouse anti-DACT3, and goat anti-β-actin (Santa Cruz Biotechnology Inc., Santa Cruz, CA).

### RT-PCR arrays

Wnt signaling RT-PCR super-arrays (PAH-043A) were obtained from SABiosciences (Frederick, MD). 1 µg of total RNA was used for reverse transcription and the entire cDNA reaction was diluted and distributed amongst the 96 wells of the super-array plate. The reactions were performed with RT^2^ SYBR Green/ROX PCR Master Mix (SABiosciences). Results were analyzed using software provided by the vendor http://www.sabiosciences.com/pcr/arrayanalysis.php.

### MicroRNA microarray analysis

Low molecular RNAs (small RNAs) were extracted from cell plates using mirVana™ miRNA isolation kit (Ambion, Austin, TX) using the miRNA enrichment method according to the manufacturer's protocol. Three hundred nanograms of small RNAs from tissue samples were directly labeled with N-code miRNA labeling kit (Invitrogen) per vendor's protocols. Briefly, mature miRNAs in the small RNA samples were first tailed with poly (A); poly (A) tailed miRNAs were then tagged with a capture sequence followed by hybridization to Cy5/Cy3 labeled 3DNA dendrimers. For microarray data processing and normalization, microarray chips were scanned using a GenePix 4000B scanner (Axon Instruments, Union City, CA) and median spot intensities were generated using GenePix 5.0 (Axon Instruments, Union City, CA). Microarray data were processed and normalized (50%) using GeneSpring (Agilent, CA). Statistic and clustering analyses were performed using GeneSpring software. Expression levels of miRNAs were subjected to 2-way analysis of variance (Anova) for CSC treated vs untreated conditions for the various cell lines.

### Ago CLIP (Identification of AGO-1-associated mRNAs)

Cells were harvested and CLIP experiments were performed using either anti-AGO family or elF2C antibodies as described (28). Briefly, cells were cross-linked with irradiation for 400 mJ/cm^2^ and additional 200 mJ/cm^2^ in Stratalinker, and lysed to generate RNA/protein (AGO) complexes [28]–[30]. After pre-clearing, 1% of each sample was saved as input fraction. Immunoprecipitation was performed using specific antibodies against either AGO family (Millipore, Billerica, MA), or elF2C (Santa Cruz Biotechnology, Inc.), or control IgG. RNA was isolated and purified from complexes, followed by PCR amplification of 3′ UTRs of miR-31 targets.

### Murine xenograft experiments

Calu-6 and H358 cells transfected with pCDH-CMV-MCS-EF1-copGFP vector control and pMiR-H31PA-1 were suspended in PBS at a concentration of 1×10^6^ cells/100 µL, and inoculated subcutaneously into contralateral flanks of athymic nude mice (10 mice per treatment group per experiment). Mice were monitored twice weekly and tumor volumes were calculated on the basis of perpendicular diameters. Approximately 21 days and 35 days later for Calu-6 and H358 experiments, respectively, mice were euthanized, and evaluated for percent tumor take, and mass of excised xenografts. Thereafter, tumor tissues were snap frozen in liquid nitrogen, and stored for future analysis. All animal procedures were approved by the National Cancer Institute Animal Care and Use Committee, and were in accordance with the NIH Guide for the Care and Use of Laboratory Animals.

### Statistical Analysis

Differences between matched samples from the same lung cancer patients, such as tumor and normal tissue were tested with the signed-rank test. Groups were compared with the Wilcoxon–Mann-Whitney test. Confidence intervals for the differences in medians between two groups were computed by the bias-corrected and accelerated bootstrapping methods. Differences in tumor volumes between opposing flanks of tumor-bearing mice were tested with the Wilcoxon-signed rank test at 5-day intervals. *P* values were adjusted for multiple testing by complete resampling [31]. Confidence intervals for the median of flank differences were computed as exact non-parametric two-tailed confidence intervals based on the Wilcoxon signed-rank test.

### Primer Sequences

Submitted as Table S1.

## Results

### Up-regulation of miR-31 in cultured cells exposed to CSC

Preliminary experiments using array techniques were performed to examine miRNA expression profiles in a panel of normal respiratory epithelia (SAEC and NHBE), immortalized human bronchial epithelial cells (HBEC) and lung cancer cells (Calu-6, H841, H1299, H1650, and H1975) cultured in normal media with or without CSC. This analysis revealed unique basal miRNA expression profiles coinciding with histology (normal vs malignant). Furthermore, lung cancer lines derived from smokers (Calu-6, H1299, H841) were readily distinguished from those from non-smokers (H1650, H1975) (M. Yang, manuscript in preparation).

**Figure 1:**
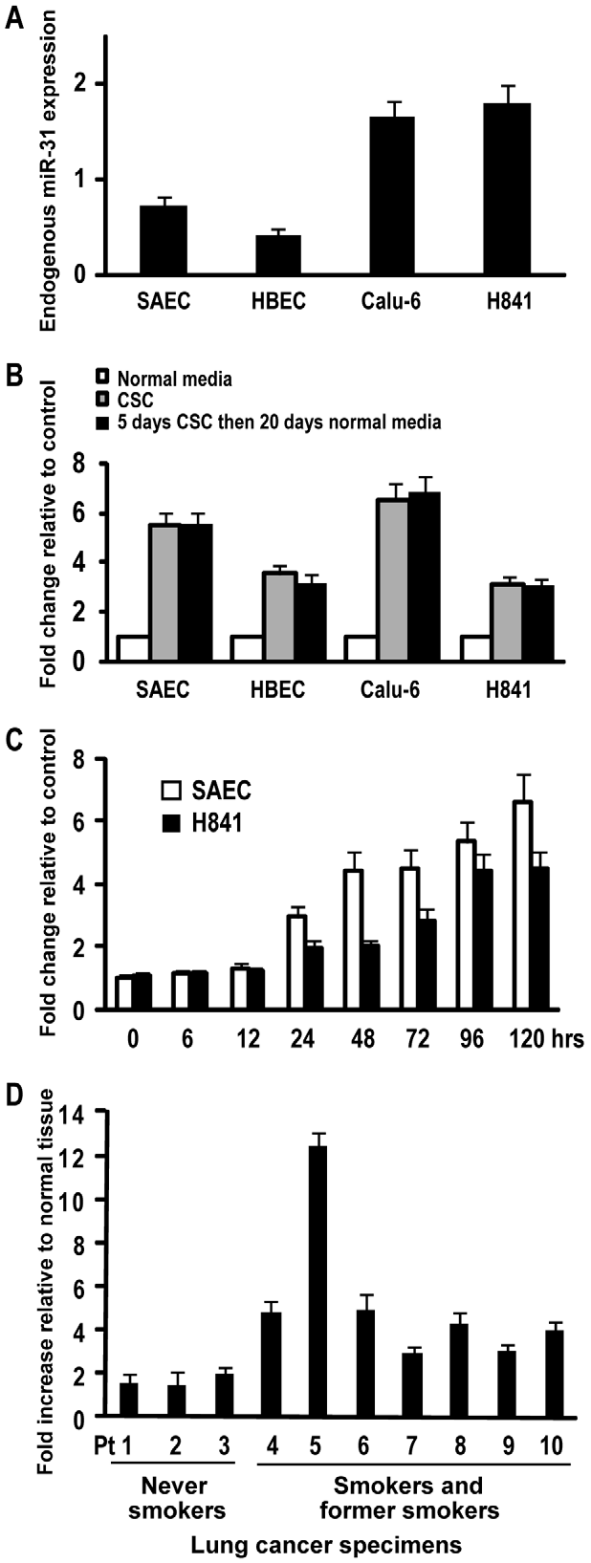
Expression of miR-31 in cultured cells exposed to CSC, primary lung cancers and adjacent normal lung tissues. A) qRT-PCR analysis of endogenous miR-31 expression normalized with control miRNA (RNU44) in SAEC, HBEC, Calu-6, and H841cells. Basal levels of miR-31 are higher in lung cancers relative to cultured normal or immortalized human respiratory epithelial cells. B) qRT-PCR analysis of miR-31 expression in SAEC, HBEC, Calu-6, and H841cells cultured in normal media (NM) with or without CSC for 5 days. Increased miR-31 expression was evident 20 days following discontinuation of CSC treatment. C) qRT-PCR analysis demonstrating time-dependent activation of miR-31 in SAEC and H841cells cultured in NM with or without CSC for 0, 12, 24, 48, 72, 96, and 120 hours. D) qRT-PCR analysis demonstrating miR-31 expression in human lung cancers relative to paired adjacent normal lung tissues. miR-31 levels in tumors were higher than corresponding normal lung. Furthermore, miR-31 levels were significantly higher in lung cancers from active/former smokers compared to those from never-smokers.

Of particular interest, aforementioned micro-array experiments suggested that CSC treatment induced expression of miR-31 in normal respiratory epithelia as well as lung cancer cells. To further investigate this phenomenon, quantitative RT-PCR (qRT-PCR) experiments were performed to examine miR-31 expression in SAEC, HBEC, Calu-6, and H841 cells cultured in normal media (NM) with or without CSC for 5 days. These experiments (Figure 1A) demonstrated that untreated Calu-6 and H841cells exhibited higher endogenous levels of miR-31 relative to SAEC and HBEC cells; additional analysis revealed that 5 day CSC exposure up-regulated miR-31 expression 5.5 fold and 3.6 fold in SAEC and HBEC, respectively relative to controls. Similar treatment increased miR-31 expression 3.1 and 6.5 fold in Calu-6 and H841 cells, respectively (Figure 1B). Time course experiments revealed up-regulation of miR-31 in SAEC and H841 cells within 24 h following initiation of CSC exposure, with expression peaking and leveling off approximately 96 h later (Figure 1C). Interestingly, up-regulation of miR-31 by CSC persisted for 20 days following removal of CSC from culture media (Figure 1B).

### miR-31 expression in primary lung cancer specimens

Additional qRT-PCR experiments were performed to examine miR-31 expression levels in a randomly selected panel of primary non-small cell lung cancers and adjacent histologically normal lung parenchyma (clinical and pathologic data summarized in Table 1). As shown in Figure 1D, miR-31 expression was increased (mean 4.13 fold; range 1.47–12.46 fold) in lung cancers relative to paired normal lung tissues. Interestingly, miR-31 levels in lung cancers from smokers were higher than those observed in non-smokers (5.2 vs 1.65 fold, respectively; p<0.01). Collectively, these data confirmed preliminary experiments demonstrating higher levels of miR-31 expression in lung cancer cells relative to cultured normal respiratory epithelia (Figure 1A), and suggested that activation of miR-31 might be a biologically-relevant phenomenon during human pulmonary carcinogenesis.

**Table 1:** Clinicopathologic characteristics of 10 lung cancer patients used for miR31 analysis*.

Characteristic		N (%)
Sex	Male	1 (10)
	Female	9 (90)
Age, y**		66.2 (46–85)
Tumor types	Adenocarcinoma	9 (90)
	Squamous cell carcinoma	1 (10)
Stage	T1–T2	8 (80)
	T3–T4	2 (20)
	N0–N1	9 (90)
	N2	1 (10)
Smoking history	Yes	7 (70)
	No	3 (30)

*All lung cancer patients underwent surgery with curative intent.

**Age is reported as mean (range).

### Effects of miR-31 on antagonists of WNT signaling

**Figure 2:**
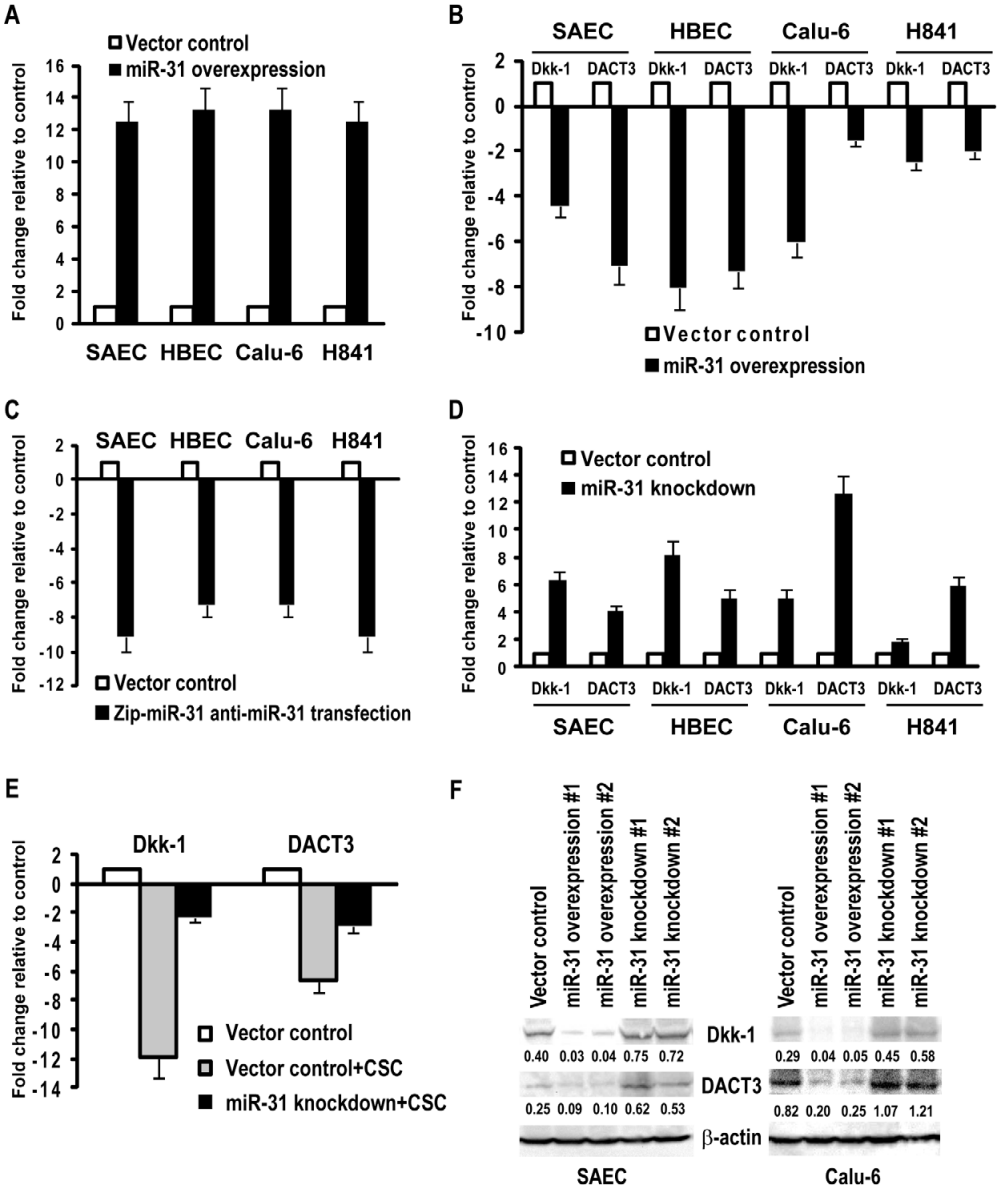
miR-31 negatively regulates WNT signaling pathway antagonists in normal respiratory epithelia and lung cancer cells. A) miR-31 was over-expressed in SAEC, HBEC, Calu-6, and H841 cells via transient transfection of primary miR-31 constructs. qRT-PCR analysis confirmed high level miR-31 expression in miR-31-transfected relative to control cells. B) qRT-PCR analysis of Dkk-1 and DACT3 expression levels in SAEC, HBEC, Calu-6, and H841 cells with or without over-expression of miR-31. Over-expression of miR-31 decreased Dkk-1 and DACT3 in all four cell lines. C) qRT-PCR analysis demonstrating decreased levels of endogenous miR-31 in SAEC, HBEC, Calu-6, and H841 cells following transient transfection of antisense-miR-31(Zip-miR-31) constructs relative to controls. D) qRT-PCR analysis of Dkk-1 and DACT3 expression levels in SAEC, HBEC, Calu-6 and H841 cells with or without down-regulation of miR-31. Knock-down of miR-31 enhances basal levels of Dkk-1 and DACT3 in these cells. E) qRT-PCR analysis demonstrating that knockdown of miR-31 partially blocks CSC-mediated decreases of Dkk-1 and DACT3 in SAEC. F) Western blot analysis of Dkk-1 and DACT3 expression in parental and vector control SAEC and Calu-6 cells, as well as SAEC and Calu-6 cells exhibiting constitutive over-expression or knock-down of miR-31. Densitometry values are normalized to b-actin control. Dkk-1 and DACT3 level were decreased, or somewhat enhanced in these cells following over-expression, or knock-down of miR-31, respectively.

Software-guided analysis demonstrated that several antagonists of Wnt signaling including Dkk-1[32] and DACT3[33] were potential miR-31 targets. To confirm these results, SAEC, HBEC, Calu-6, and H841 cells were transiently transfected with miR-31. Quantitative RT-PCR experiments revealed ∼12 fold increases in miR-31 expression in miR-31-transfected cells relative to vector controls (Figure 2A). Over-expression of miR-31 decreased Dkk-1 as well as DACT3 (∼5–8 fold and ∼1.5–7 fold, respectively, relative to controls) in these four cell lines (Figures 2B). These effects appeared somewhat more pronounced in normal SAEC and immortalized HBEC, possibly due to lower levels of endogenous miR-31 and higher levels of Dkk-1 and DACT3 in these cells relative to Calu-6 and H841 lung cancer cells.

Subsequent experiments were undertaken to determine if depletion of endogenous miR-31 affected expression of Dkk-1 and DACT3 in cultured respiratory epithelial cells. As shown in Figure 2C, miR-31 expression levels were reduced ∼7–10 fold in SAEC, HBEC, Calu-6, and H841 cells transiently transfected with antisense-miR-31 (Zip-miR-31) constructs relative to vector controls. Reduction of miR-31 expression increased Dkk-1 as well as DACT3 expression in these cell lines (∼1.47–8 fold and ∼4.7–9 fold respectively; Figure 2D). Subsequent experiments revealed that knockdown of miR-31 significantly attenuated CSC-mediated decreases in Dkk-1 and DACT3 in SAEC as well as H841 cells (Figure 2E). Consistent with aforementioned results, western blot analysis revealed that Dkk-1 and DACT3 protein levels in SAEC and Calu-6 cells were decreased by over-expression of miR-31 (Figure 2F). In contrast, knock-down of endogenous miR-31 appeared to increase Dkk-1 and DACT3 protein levels in SAEC and Calu-6 cells, although these changes did not correlate precisely with alterations in mRNA expression, possibly due, in part, to protein stability and antibody affinities. Collectively, these experiments strongly suggested that miR-31 modulates Dkk-1 and DACT3 via post-transcriptional and translational-inhibitory mechanisms in cultured normal respiratory epithelia and lung cancer cells.

Super-arrays were used to further examine the effects of miR-31 on Wnt signaling in SAEC and Calu-6 cells. This analysis revealed 7–14 fold down-regulation of Dkk-1, as well as additional antagonists of Wnt signaling including SFRP1, SFRP4, and WIF1, as well as a 5 fold increase in CTNNB1, TCF7 and Wnt-5a (data available on request). Subsequent qRT-PCR experiments confirmed repression of SFRP1, SFRP4, and WIF-1, and up-regulation of Wnt-5a in SAEC and Calu-6 cells over-expressing miR-31 (Figure S1A). Collectively, these data suggest that miR-31 activates Wnt signaling in cultured lung cancer and normal respiratory epithelial cells.

### Dkk-1 and DACT3 are direct target candidates of miR-31

**Figure 3:**
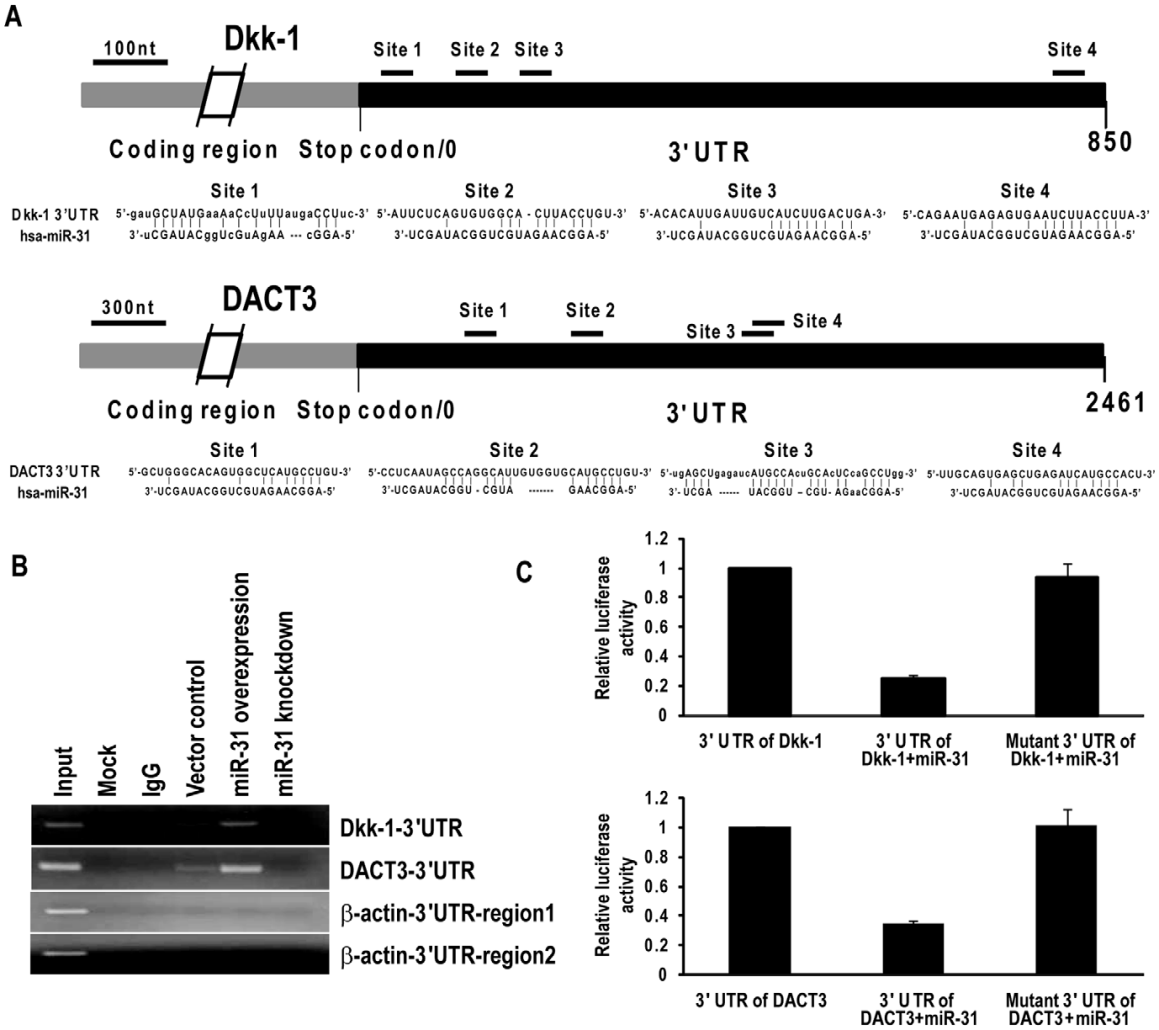
WNT signaling pathway antagonists are direct target candidates of miR-31. A) Putative target sites of miR-31 within Dkk-1 3′ UTR (top) and DACT3 3′ UTR (bottom). B) miRNA-targeted transcripts in RISC were precipitated with AGO family antibodies after UV induced cross-linking of RNAs to their binding proteins, followed by RT-PCR amplification. Over-expression of miR-31 significantly increased precipitation of Dkk-1 and DACT3 target transcripts in SAEC cells. β-actin served as the negative control since no sequences in the 3′ UTR that can be targeted by miR-31. C) Luciferase assays demonstrating reduction in luciferase activity in SAEC transfected with pMiR-Report vectors with or without insertion 3′ UTR or mutated 3′ UTR of WNT signaling antagonists.

Additional experiments were undertaken to examine if miR-31 directly interacts with 3′ UTRs of Dkk-1 and DACT3. Briefly, RNA cross-link immunoprecipitation (CLIP) techniques were used to detect interaction of miR-31 with these potential targets in SAEC cells [28]. Figure 3A depicts the potential miR-31 binding sites within the 3′ UTRs of Dkk-1 and DACT3 transcripts. RT- PCR analysis revealed that the AGO family antibody precipitated Dkk-1 and DACT3 transcripts in the cytoplasm of untreated SAEC (Figure 3B). Obvious increases in PCR products corresponding to 3′ UTRs of Dkk-1 and DACT3 were observed in SAEC over-expressing miR-31; this phenomenon was not observed in miR-31-depleted SAEC cells. In these experiments, β-actin served as the negative control since no sequences can be targeted by miR-31 (Figure 3B). Because miR-21 has been found to directly target the 3′ UTR of PDCD4 human tissues [34], immunoprecipitated RNA from SAEC cells over-expressing miR-21 served as a positive control for CLIP (data not shown). Consistent with aforementioned Wnt SuperArray results, additional CLIP experiments demonstrated interaction of miR-31 with SFRP4 (Figure S1B).

Additional experiments were performed using pMiR-Report vectors with wt or mutant 3′ UTRs of Dkk-1 or DACT3, transiently co-transfected with miR-31 primary constructs or control vectors into SAEC. As shown in Figure 3C, luciferase activities of wt 3′ UTRs of Dkk-1 and DACT3 were reduced ∼70% compared with vector control or mutated 3′ UTRs, respectively. Collectively, these experiments strongly suggest that miR-31 directly interacts with 3′ UTRs of Dkk-1 and DACT3.

### Epigenetic alterations coinciding with CSC-mediated activation of miR-31

**Figure 4:**
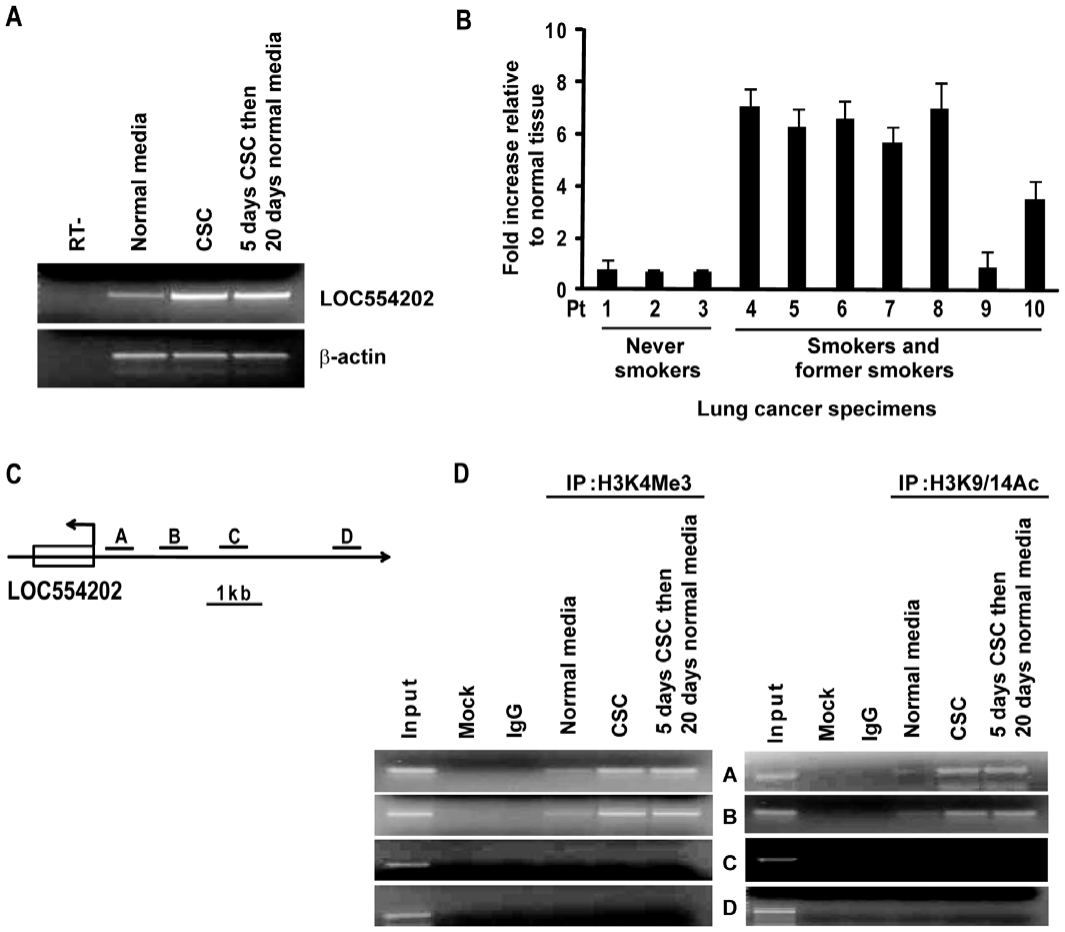
Epigenetic alterations coinciding with CSC-mediated activation of miR-31. A) RT-PCR analysis demonstrating up-regulation of LOC554202 expression in SAEC following 5-day CSC exposure. Consistent with data pertaining to up-regulation of miR-31 by CSC, expression of LOC554202 persisted for 20 days following cessation of CSC exposure. B) qRT-PCR analysis demonstrating expression levels of LOC554202 in primary lung cancers relative to adjacent normal lung tissues. C) Schematic representation of LOC554202, the putative host gene of miR-31. A, B, C, and D represent positions of paired primers used for ChIP analysis. D) ChIP analysis depicting H3K4Me3 and H3K9/14 acetylation levels within four regions of the LOC554202 promoter (∼1 kb, 2 kb, 3 kb, and, 5 kb from the TSS) in SAEC cultured with or without CSC. Increased activation marks were observed in the proximal promoter region following CSC exposure.

Recent ChIP analysis of distribution patterns of H3K4Me3, H3K9/14Ac, and H2AZ suggested that the host gene for miR-31 is LOC554202 [35]. As such, additional experiments were performed to examine if CSC modulated expression of LOC554202 in SAEC. Consistent with results pertaining to miR-31 (Figure 1A), 5-day CSC treatment induced expression of LOC554202, which was maintained for 20 days following removal of CSC from culture media (Figure 4A); these results were consistent with those pertaining to CSC-mediated up-regulation of miR-31 (Figure 1). qRT-PCR experiments revealed that 6 of 7 primary lung cancer specimens expressing ≥2 fold higher miR-31 levels relative to paired normal lung tissues also exhibited over-expression of LOC554202 (Figure 4B).

Sequence analysis revealed no classic CpG island in the promoter region of LOC554202, suggesting that this host gene is not regulated primarily via DNA methylation mechanisms (data not shown). Subsequent ChIP experiments demonstrated increased levels of H3K4Me3 and H3K9/14Ac activation marks in the proximal promoter region (0 to −1.5 k) of LOC554202 (which presumably contains the regulatory elements for miR-31) in SAEC following CSC exposure (Figures 4C and D). Interestingly, these activation marks in the LOC554202 promoter region in SAEC were still present 20 days following cessation of CSC treatment. These findings were consistent with RT-PCR results depicted in Figure 4A.

### Role of C/EBP-β in CSC-mediated activation of miR-31

**Figure 5:**
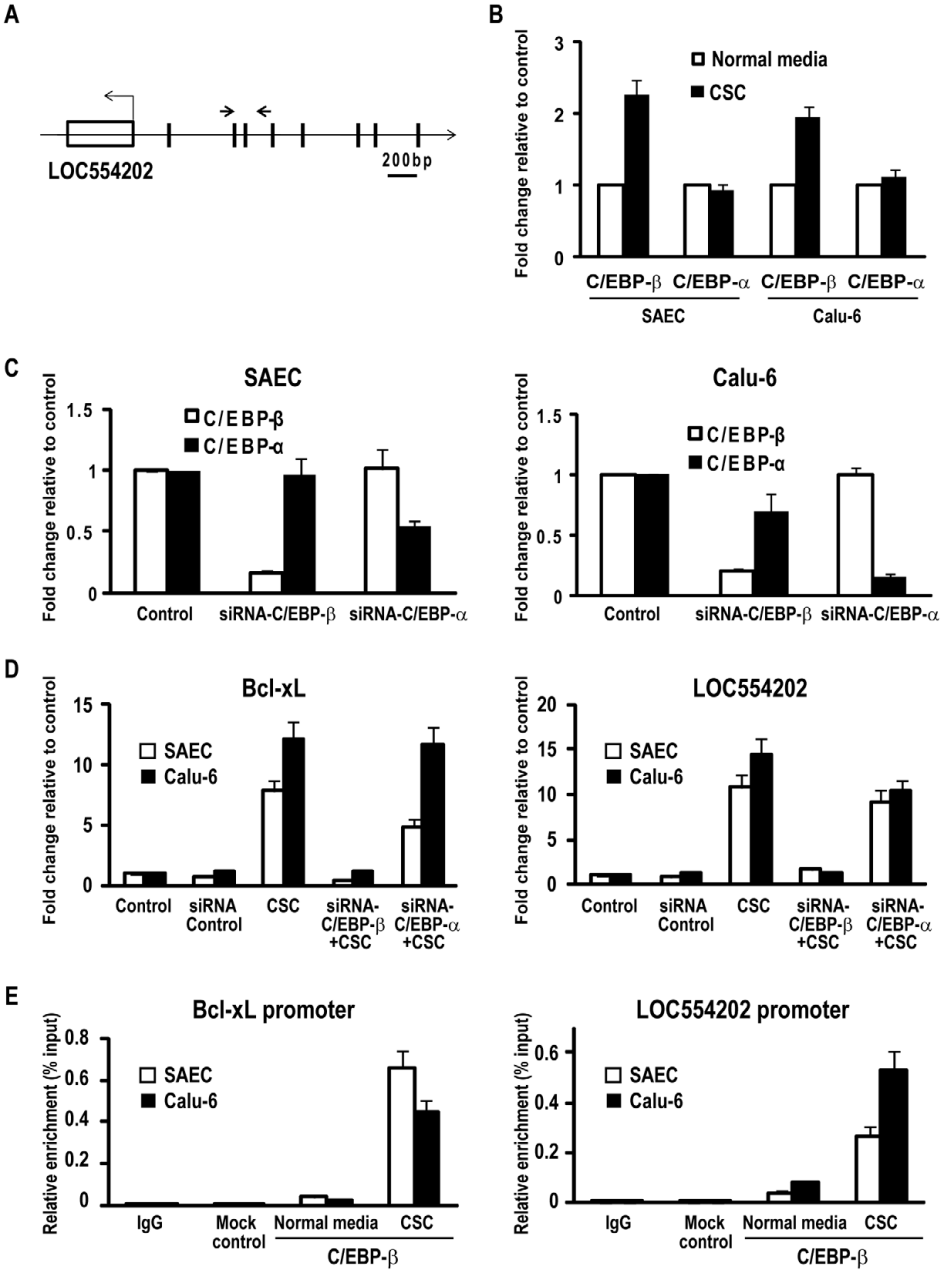
C/EBP-β mediates CSC-induced miR-31 transcription. A) Schematic representation of putative binding sites of C/EBP-β (vertical bars) in the LOC554202 promoter. The arrows show the positions of ChIP primers for C/EBP-β. B) qRT-PCR analysis demonstrating that CSC enhances C/EBP-β but not C/EBP-α expression in SAEC and Calu-6 cells. C) qRT-PCR analysis demonstrating specificity of siRNAs targeting C/EBP-β and C/EBP-α in SAEC and Calu-6 cells. D) Left panel: qRT-PCR analysis of Bcl-xL expression in SAEC and Calu-6 cells cultured in the presence or absence of CSC following knock-down of C/EBP-β or C/EBP-α. CSC exposure dramatically increases Bcl-xL expression in SAEC and Calu-6 cells. Knock-down of C/EBP-β expression decreases activation of Bcl-xL by CSC. This phenomenon was not nearly as dramatic following knock-down of C/EBP-α. Right panel: qRT-PCR analysis of LOC554202 expression in SAEC and Calu-6 cells cultured in the presence or absence of CSC following knock-down of C/EBP-β or C/EBP-α. CSC exposure dramatically increases LOC554202 expression in SAEC and Calu-6 cells. Knock-down of C/EBP-β expression virtually abolishes activation of LOC554202 by CSC in SAEC as well as Calu-6 cells. This phenomenon was considerably less apparent following knock-down of C/EBP-α. E) Left panel: ChIP analysis demonstrating enrichment of C/EBP-β within the Bcl-xL promoter in SAEC and Calu-6 cells following CSC exposure. Right panel: ChIP analysis demonstrating enrichment of C/EBP-β in the LOC554202 promoter in SAEC and Calu-6 cells following CSC exposure.

Additional experiments were performed to further examine mechanisms by which CSC mediates activation of LOC554202. Preliminary software analysis revealed no characteristic XRE sequences within 5 kb of the presumed transcription start site (TSS) for LOC554202, suggesting that up-regulation of LOC554202 by CSC was not mediated primarily via aryl hydrocarbon receptor signaling [36]. However, as shown in Figure 5A, this analysis revealed multiple potential binding sites for C/EBP-β, which has been shown previously to mediate up-regulation of Bcl-xL in breast cancer cells exposed to cigarette smoke [37]. As such, additional experiments were performed to ascertain if this transcription factor contributed to CSC-mediated activation of miR-31 in cultured respiratory epithelia. qRT-PCR experiments revealed that CSC enhanced C/EBP-β - but not CEBP/α expression in SAEC as well as Calu-6 cells (Figure 5B). In additional experiments, siRNA techniques were used to inhibit C/EBP expression in these cells. Preliminary experiments confirmed specificity of siRNAs targeting C/EBP-β and C/EBP-α (Figure 5C). Control experiments demonstrated up-regulation of Bcl-xL expression in SAEC and Calu-6 cells by CSC exposure, and complete abrogation of CSC-mediated induction of Bcl-xL expression in these cells following knock-down of C/EBP-β (Figure 5D; left panel). Knockdown of C/EBP-β also markedly attenuated CSC-mediated activation of LOC554202 in these cells; this phenomenon was considerably less pronounced following knock-down of CEBP-α (Figure 5D; right panel).

Quantitative ChIP experiments were next performed to examine if CSC induces recruitment of C/EBP-β to the LOC554202 promoter. Preliminary control experiments revealed increased occupancy of C/EBP-β within the Bcl-xL promoter in SAEC and Calu-6 cells following CSC exposure (Figure 5E; left panel). Subsequent experiments demonstrated enrichment of C/EBP-β within the promoter region of LOC554202 in SAEC as well as Calu-6 cells following CSC treatment (Figure 5E; right panel). Collectively, these data strongly suggest that C/EBP-β contributes to up-regulation of LOC554202 and miR-31 by CSC.

### Effects of miR-31 on proliferation and tumorigenicity of human lung cancer cells

**Figure 6:**
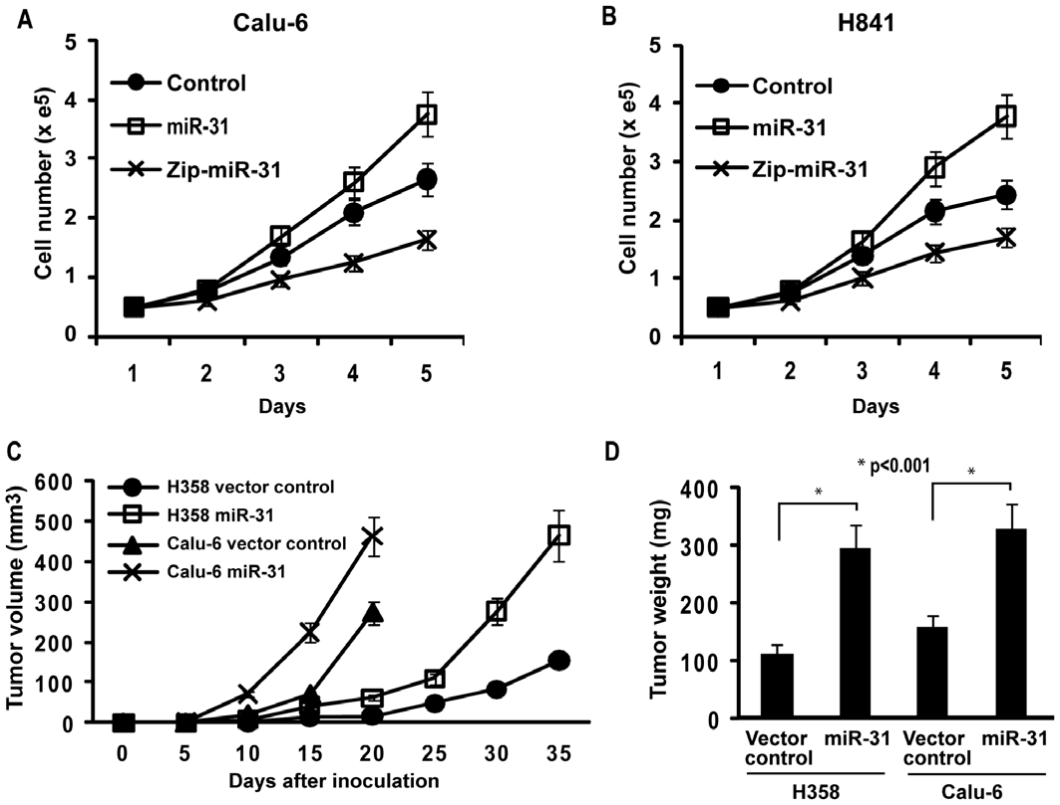
miR-31 functions as an oncomir in lung cancer cells. A/B) Direct cell count assays depicting in-vitro proliferation of Calu-6 and H841 lung cancer cells exhibiting over-expression or knock-down of miR-31. Constitutive over-expression of miR-31 increased proliferation, whereas knock-down of miR-31 diminished proliferation of Calu-6 (a) as well as H841 (b) cells relative to vector controls. C) Growth of H358 and Calu-6 tumor xenografts in nude mice. Calu-6 and H358 cells constitutively over-expressing miR-31 and vector controls (1×10^6^) were inoculated subcutaneously in the right and left flanks of athymic nude mice. Tumor volumes were determined every five days. The mean tumor volumes are shown. Volumes of xenografts derived from Calu-6 or H358 cells over-expressing miR-31 were significantly larger than vector controls (P<0.01). D) Tumor masses from H358 and Calu-6 xenografts. MiR-31 expression significantly increased average mass of H358 as well as Calu-6 xenografts (p<0.001).

Additional experiments were undertaken to ascertain if miR-31 enhances the malignant phenotype of lung cancer cells. First, in-vitro growth assays were performed using Calu-6 and H841 cells exhibiting over-expression or depletion of miR-31. As shown in Figures 6A and B, constitutive over-expression of miR-31 increased proliferation of Calu-6 (35%) and H841(55%) relative to vector controls; in contrast, knock-down of endogenous miR-31 mediated ∼30% growth inhibition of these cancer cells. Additionally, Calu-6 as well as H358 cells stably-transfected with miR-31 or vector controls were inoculated subcutaneously in contralateral flanks of athymic nude mice (10 per group). As shown in Figures 6C and D, constitutive over-expression of miR-31 markedly increased volumes and masses of H358 as well as Calu-6 xenografts relative to control vectors (p<0.01). Collectively, these experiments strongly suggest that miR-31 enhances the malignant phenotype of human lung cancer cells.

## Discussion

The vast majority of lung cancers are directly attributable to cigarette abuse [38]; furthermore, a substantial number of lung cancers in never-smokers are due to environmental tobacco exposure (passive smoking) [39]. An estimated 1.3 billion people smoke worldwide, and the prevalence of smoking, as well as environmental tobacco exposures are increasing at an alarming rate [40]. The overall five-year survival rate for lung cancer patients is approximately 15 percent [41]. These data warrant continued efforts to define genetic as well as epigenetic mechanisms contributing to pulmonary carcinogenesis, and to identify novel molecular targets for the treatment and prevention of lung cancer.

In the present study, an in-vitro model system was used to gain insight regarding miRNA alterations potentially contributing to initiation and early progression of tobacco-induced lung cancers. Our analysis revealed consistent activation of miR-31 in normal respiratory epithelia and lung cancer cells derived from smokers as well as never-smokers. Subsequent analysis revealed over-expression of miR-31 in primary human lung cancer specimens –particularly those from smokers, relative to adjacent normal parenchyma. Interestingly, up-regulation of miR-31 has been observed in pulmonary adenomas or normal lung tissues from mice exposed to vinyl carbamate, or environmental smoke, respectively [42], [43].

Until recently, limited information has been available regarding mechanisms modulating miRNA expression during malignant transformation, primarily due to difficulties pertaining to identification of promoters and transcription start sites (TSS). RNA polymerase II (RNAPII) appears to regulate several miRNAs including miR-21 and miR-17, whereas RNA polymerase III (RNAPIII) has been implicated in the regulation of C19MC and other miRNAs within or adjacent to DNA repeats [44]. Upstream sequencing of miRNAs by RNAPII ChIP-chip techniques revealed that LOC554202, which maps to 9p21.3, shares a TSS with miR-31, and acts as its host gene [35]. Consistent with these observations, qRT-PCR and ChIP experiments presented in this manuscript demonstrated that CSC-mediated up-regulation of miR-31 coincides with activation of LOC554202.

Whereas several reports have documented up-regulation of miR-31 in cultured lines or tissues derived from oropharyngeal, esophageal, lung, or colorectal carcinomas [45]–[48], our experiments appear to be the first to directly examine mechanisms regulating expression of this miRNA and its host gene in human lung cancer. Notably our analysis revealed a critical role for C/EBP-β in activation of miR-31 and LOC554202 by cigarette smoke. C/EBP-β, which exists as several isoforms exhibits highly pleiotropic roles in normal tissue homeostasis and cancer. Recent studies indicate that C/EBP-β is a master regulator of mesenchymal transformation and aggressive phenotype of human glioma cells [49]. In addition, C/EBP-β activates a variety of pro-survival and pro-metastatic genes, enhancing resistance to apoptosis in multiple myeloma and prostate carcinoma cells [50], [51]. Furthermore, C/EBP-β regulates energy balance, and promotes growth of colon cancer cells in part via activation of IGF-1, insulin, and leptin [52]. Lastly, C/EBP-β activates Bcl-xL expression in breast and lung cancer cells exposed to CSC [37] (and our current experiments). The specific isoforms of C/EBP-β activating miR-31 and LOC554202, and the mechanisms mediating prolonged up-regulation of this miRNA and its host gene in respiratory epithelia and lung cancer cells following CSC exposure are a focus of ongoing investigation in our laboratory.

Shortly before this manuscript was initially submitted for publication, Liu et al [47] reported a series of experiments demonstrating up-regulation of miR-31 in lung cancer cells derived from cyclin E-transgenic mice. Subsequent experiments revealed over-expression of miR-31 in human lung adenocarcinomas. Knock-down of miR-31 inhibited proliferation of mouse and human lung cancer cells, and diminished clonogenicity and tumorigenicity of murine lung cancer cells. The oncogenic effects of miR-31 in lung cancer cells were attributed to inhibition of large tumor suppressor 2 (LATS2) and PP2A regulatory subunit B alpha (PPP2R2A).

Our current data are consistent with, and extend these findings by demonstrating an additional potential mechanism by which miR-31 contributes to pulmonary carcinogenesis, namely activation of Wnt signaling. Our previous studies have demonstrated that CSC induces polycomb-mediated repression of Dkk-1 in lung cancer cells and cultured normal respiratory epithelia [32]. CSC-exposure as well as Dkk-1 knock-down enhances Wnt-5a expression, and significantly increases proliferation and tumorigenicity of lung cancer cells. Our current data indicate that miR-31 directly inhibits expression of Dkk-1 and DACT3 in lung cancer cells as well as normal respiratory epithelia; furthermore, miR-31 over-expression in these cells depletes several other antagonists of Wnt signaling with potential miR-31 binding motifs including SFRP1, SFRP4, and WIF-1, which are frequently silenced in human lung cancers [53]. In addition, up-regulation of miR-31 mediates expression of the non-canonical ligand Wnt5a, which induces epithelial-to-mesenchymal transition (EMT), and dramatically enhances the malignant phenotype of lung cancer cells (Ripley et al, submitted for publication). Collectively, these observations suggest that miR-31 cooperates with epigenetic mechanisms to silence Wnt antagonists, thereby activating signaling networks implicated in maintenance of normal as well as cancer stem cells [54], [55] during initiation and progression of tobacco-induced lung cancers. These issues are a focus of ongoing experiments in our laboratory.

Our findings, as well as those of Liu et al [47] are consistent with data demonstrating that up-regulation of miR-31 in oropharyngeal and colorectal carcinomas correlates with advanced stage of disease, and decreased survival of patients with these malignancies [45], [48]. On the other hand, our data differ from those reported by Valastyan et al [56], Creighton et al [57], and Ivanov and colleagues [58], who have observed that miR-31 functions as a tumor suppressor in breast and ovarian carcinomas, as well as malignant pleural mesotheliomas. Discrepancies regarding our findings and those of the latter investigators may be attributable in part to genetic and epigenetic profiles of the various malignancies, reprogramming [59], tissue-specific effects of basal and inducible miR-31 expression, and Wnt signaling [60], [61]. Further studies are necessary to fully define the role of miR-31 in human malignancies, and specifically determine the prognostic and predictive significance of miR-31 expression during pulmonary carcinogenesis.

## Supporting Information

Figure S1 A) qRT-PCR analysis demonstrating that over-expression of miR-31 decreases SFRP1, SFRP4 and WIF-1, and enhances Wnt5a expression in SAEC and Calu-6 cells. B) CLIP analysis revealing interaction of miR31 with 3′ UTR of SFRP4.
(0.12 MB TIF)

Table S1 Primer sequences for amplification, qRT-PCR, ChIP, CLIP, and site-directed mutagenesis
(0.07 MB DOC)
