# Supplementary figures and images for: Cigarette Smoke Induces C/EBP-β-Mediated Activation of miR-31 in Normal Human Respiratory Epithelia and Lung Cancer Cells
Source: PLoS One. 2010 Oct 29;5(10):e13764. doi: 10.1371/journal.pone.0013764 (PMC2966442; doi:10.1371/journal.pone.0013764)

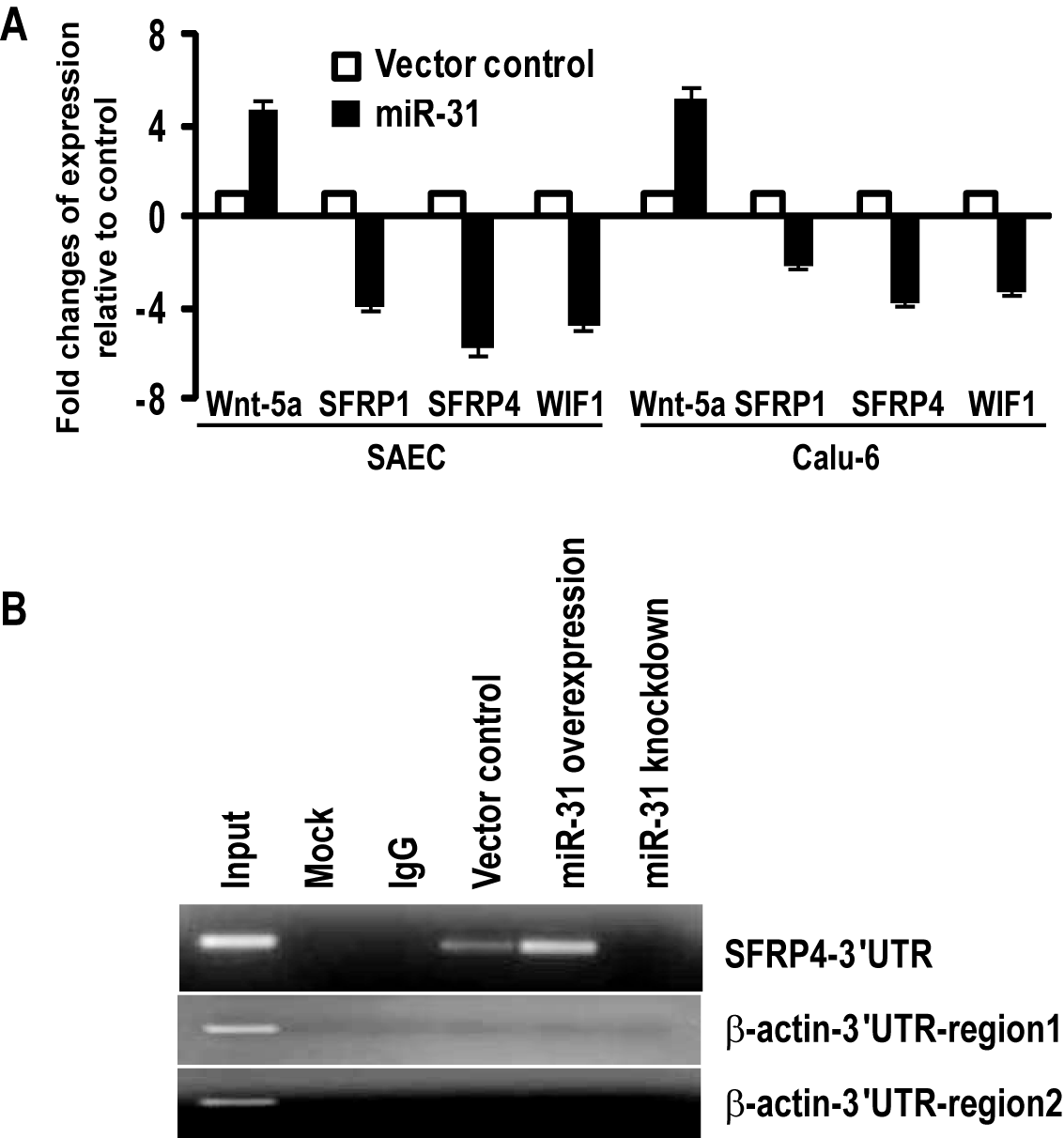

Supplement: Figure S1 — A) qRT-PCR analysis demonstrating that over-expression of miR-31 decreases SFRP1, SFRP4 and WIF-1, and enhances Wnt5a expression in SAEC and Calu-6 cells. B) CLIP analysis revealing interaction of miR31 with 3′ UTR of SFRP4. (0.12 MB TIF) [file pone.0013764.s001.tif]
