# Supplementary material for: Cigarette Smoke Induces C/EBP-β-Mediated Activation of miR-31 in Normal Human Respiratory Epithelia and Lung Cancer Cells
Source: PLoS One. 2010 Oct 29;5(10):e13764. doi: 10.1371/journal.pone.0013764 (PMC2966442; doi:10.1371/journal.pone.0013764)
Supplement: Table S1 — Primer sequences for amplification, qRT-PCR, ChIP, CLIP, and site-directed mutagenesis (0.07 MB DOC) [file pone.0013764.s002.doc]

Table S1: Primer sequences for amplification, qRT-PCR, ChIP, CLIP, and site-directed mutagenesis

| **Name** | **Position** | **Sequence** |
| --- | --- | --- |
| **Amplification of 3' UTR of target sequences** | | |
| Dkk-1 | 3' UTR | F: 5'-AAGCTTACCAGCTATCCAAATGCAGTG-3' |
|  |  | R: 5'-ACTAGTTTCACTCTCATTCTGCCATGA-3' |
| DACT3 | 3' UTR (0-800bp) | F: 5'-AAGCTTTGGTTGTGGGCACAGACTT-3' |
|  |  | R: 5'-ACTAGTCCTGGCTATTGAGGCACTGT-3' |
|  | 3' UTR (800-1600bp) | F: 5'-AAGCTTGCCTCTGCTCTAAGGCATTG-3' |
|  |  | R: 5'-ACTAGTCCACTCCCCCACTTAGTTCA-3' |
|  | 3' UTR (1600-2400bp) | F: 5'-AAGCTTGAGAAGGGTCAGCTCAGTGG-3' |
|  |  | R: 5'-ACTAGTGAGTGCAGTAGCCCGATCTC-3' |
| **ChIP** | | |
| LOC554202 | Promoter 1kb | F: 5'-GCAGTGGAAAGGTTCAGAGC-3' |
|  |  | R: 5'-TATCCTCAACCCTCCGTGTC-3' |
|  | Promoter 2kb | F: 5'-TTTCTTGGTCAAGGCAGTCA-3' |
|  |  | R: 5'-TTGCATGAAGATTTGAGTCCA-3' |
|  | Promoter 3kb | F: 5'-AATGCCAGAGAGACCGCTAA-3' |
|  |  | R: 5'-TGCATGTAAGCAAACCCAAA-3' |
|  | Promoter 5kb | F: 5'-CAGGATGCCCTAGCACTTTC-3' |
|  |  | R: 5'-GATCAGCATCAGCAAGACCA-3' |
|  | Promoter 1-0.6kb | F: 5'-CCCTTCAAATCCAGGTGAAA-3' |
|  |  | R: 5'-ATGAGAGCGCCTTGTGAAAT-3' |
| Bcl-xL | Promoter 1kb | F: 5'-CGGGTGGCAGGAGGCCGCGGC-3' |
|  |  | R: 5'-AACTCAGCCGGCCTCGCGGTG-3' |
| **CLIP** | | |
| Dkk-1 | 3' UTR (150-300bp) | F: 5'-TCTCAGTGTGGCACTTACCTG-3' |
|  |  | R: 5'-GAGGAAAAATAGGCAGTGCAG-3' |
|  | 3' UTR (300-450bp) | F: 5'-TTTTTCTAAAGGTGCTGCACTG-3' |
|  |  | R: 5'-TGTCAGTCAAGATAACAATCAATGTG-3' |
|  | 3' UTR (700-850bp) | F: 5'-GCTCTAGAATAACTTTAAAGAAAGACG-3' |
|  |  | R: 5'-AATGTAAGATTCACTCTCATTCTGC-3' |
|  | 3' UTR (350-500bp) | F: 5'-CAGTCTGGGTGACAGAGCAA-3' |
|  |  | R: 5'-TGTTGGGATTACAGGCATGA-3' |
|  | 3' UTR (1350-1500bp) | F: 5'-AGGAGAATCGCTTGAACCTG-3' |
|  |  | R: 5'-GACGAAGTCTCGCTCTGTCA-3' |
|  | 3' UTR (50-200) | F: 5'-GATGAGATTGGCATGGCTT-3' |
|  |  | R: 5'-CACCTTCACCGTTCCAGTTT-3' |
|  | 3' UTR (300-450) | F: 5'-AAAAGCCACCCCACTTCTCTCT-3' |
|  |  | R: 5'-ATGCTATCACCTCCCCTGTG-3' |
| **Expression of LOC554202 & Bcl-xL** | | |
| LOC554202 |  | F: 5'-TGCCAGTAGAGGGAAGAGGA-3' |
|  |  | R: 5'-TCCTGTCAGATCAGCAGTGG-3' |
| Bcl-xL |  | F: 5'-TTGGACAATGGACTGGTTGA-3' |
|  |  | R: 5'-GTAGAGTGGATGGTCAGTG-3' |
| GAPDH |  | F: 5'-GGGAAGCCACTGGCATGGCCTTCC-3' |
|  |  | R: 5'-CATGTGGGCCATGAGGTCCACCAC-3' |
| β-actin |  | F: 5'-TCACCCACACTGTGCCCATCTACGA-3' |
|  |  | R: 5'-CAGCGGAACCGCTCATTGCCAATGG-3' |
| **Site-Directed Mutagenesis** | | |
| miR-31-Dkk-1 | pattern 201-249 | F: 5'-GTAAATTCTCAGTGTGGCACGTAAATGCAATG-3' |
|  |  | R: 5'-CATTGCATTTACGTGCCACACTGAGAATTTAC-3' |
|  | pattern 301-350 | F: 5'-GTACACATTGATTGTTACTGACAAATATTCTATATTG-3' |
|  |  | R: 5'-CAATATAGAATATTTGTCAGTAACAATCAATGTGTA-3' |
|  | pattern 780-850 | F: 5'-GAATGAGAGTGAATCTTCTACTTTCAAAAATAGTTTCC-3' |
|  |  | R: 5'-GGAAACTATTTTTGAAAGTAGAAGATTCACTCTCATTC-3' |
| miR-31-DACT3 | pattern 1450-1500 | F: 5'-GCAGTGAGCTGAGATCATGCCACTGCACTCGGG-3' |
|  |  | R: 5'-CCCGAGTGCAGTGGCATGATCTCAGCTCACTGC-3' |
|  | pattern 740-800 | F: 5'-GCCTCAATAGCCAGGCATTGTGGTGCGTATCCCAGC-3' |
|  |  | R: 5'-GCTGGGATACGCACCACAATGCCTGGCTATTGAGGC-3' |
|  | pattern 1890-1950 | F: 5'-GGAGAAGGGTCAGCTCAGTGGTTCATAATCCCAGC-3' |
|  |  | R: 5'-GCTGGGATTATGAACCACTGAGCTGACCCTTCTCC-3' |
|  | pattern 1451-1500 | F: 5'-CAGAGATTGCAGTGAGCTGAGATACTGCACTCCAGC-3' |
|  |  | R: 5'-GCTGGAGTGCAGTATCTCAGCTCACTGCAATCTCTGC-3' |
|  | pattern 401-450 | F: 5'-GGCTGGGCACAGTGGCTCGTAATCCCAACACTTTGGG-3' |
|  |  | R: 5'-CCCAAAGTGTTGGGATTACGAGCCACTGTGCCCAGCC-3' |
